# Risk for Transmission of Pandemic (H1N1) 2009 Virus by Blood Transfusion

**DOI:** 10.3201/eid1604.091795

**Published:** 2010-04

**Authors:** Chieko Matsumoto, Rieko Sobata, Shigeharu Uchida, Takao Hidaka, Syunya Momose, Satoru Hino, Masahiro Satake, Kenji Tadokoro

**Affiliations:** Japanese Red Cross Society Blood Service Headquarters, Tokyo, Japan

**Keywords:** Viremia, incubation period, pandemic (H1N1) 2009, influenza, transfusion, PCR, viruses, blood, expedited, letter

**To the Editor:** Influenza A pandemic (H1N1) 2009 virus emerged in early 2009 in Mexico and has since spread worldwide. In Japan, the first outbreak of the novel influenza was reported in May 2009 (*1*) and became pandemic in November. Although no cases of transfusion-transmitted influenza have been published, evidence exists of brief viremia before onset of symptoms (*2**,**3*). The possibility of transmission of this virus through transfusion of donated blood is of concern. The Japanese Red Cross Blood Centers have intercepted blood products with accompanying postdonation information indicating possible pandemic (H1N1) 2009 infection and attempted to identify the viral genome in those products by using nucleic acid amplification technology (NAT).

During June–November 2009, blood samples were collected from plasma and erythrocyte products that had been processed from donations; postdonation information indicated diagnosis of pandemic (H1N1) 2009 infection soon after donation. Viral RNA was extracted from plasma samples and erythrocyte fractions by using a QIAamp Virus Biorobot MDx kit (QIAGEN, Valencia, CA, USA) and a High Pure Viral Nucleic Acid Large Volume kit (Roche Diagnostics, Indianapolis, IN, USA), respectively. RNA samples were subjected to real-time reverse transcription–PCR (RT-PCR) of hemagglutinin (HA) and matrix (M) genes of influenza A by using PRISM 7900 (Applied Biosystems, Foster City, CA, USA). The RT-PCR of HA was specific for pandemic (H1N1) 2009 virus, whereas the RT-PCR of M was designed to detect both pandemic (H1N1) 2009 and seasonal influenza A viruses. The sequences of probes and primers were synthesized according to the protocols developed by the Japanese National Institute of Infectious Diseases (*4*). Either 200 μL of a plasma sample or 100 µL of packed erythrocytes was used for each test, and the test was performed 2× for each gene in each sample. Before the investigation using donated blood samples, the sensitivity of the NAT system was checked by spiking experiments. Viral particles of pandemic (H1N1) 2009 virus (A/California/04/2009 [H1N1]), donated by the National Institute of Infectious Diseases, were spiked into plasma and erythrocyte samples from healthy volunteers. Viral RNA was detected in the plasma samples spiked with viral particles corresponding to 300 genome equivalents/mL and in the packed erythrocyte samples spiked with viral particles corresponding to 3,000 genome equivalents/mL.

NAT was conducted by using 96 plasma and 67 erythrocyte samples obtained from 96 blood donors who had symptoms of influenza within 7 days postdonation. For 20 donors, pandemic (H1N1) 2009 was diagnosed within 1 day postdonation and, for another 20, within 2 days postdonation (Figure). Pandemic (H1N1) 2009 virus was not found in any of the samples tested, but it was consistently detected in the external positive control. These results suggest that the viremia with pandemic (H1N1) 2009 virus, if any, is very low and can be missed by current NAT or that the viremic period is too brief to identify viremia. Although the risk for transmission of pandemic influenza by transfusion seems to be low, further investigation is needed to elucidate this risk.

**Figure:**
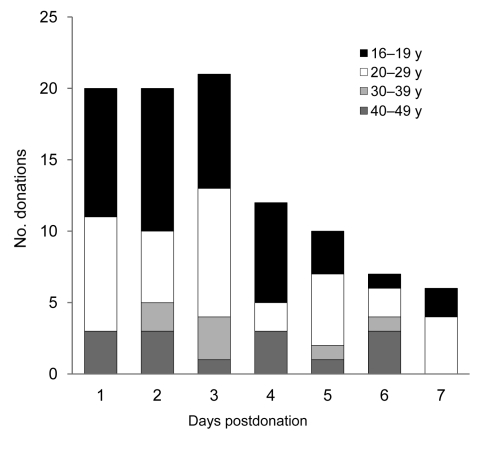
Number of blood donations from persons for whom pandemic (H1N1) 2009 infection was diagnosed postdonation and time between donation and diagnosis, by donor age, Japan.
